# Loss of the SWI/SNF-ATPase subunit members SMARCF1 (ARID1A), SMARCA2 (BRM), SMARCA4 (BRG1) and SMARCB1 (INI1) in oesophageal adenocarcinoma

**DOI:** 10.1186/s12885-019-6425-3

**Published:** 2020-01-06

**Authors:** Simon Schallenberg, Julian Bork, Ahlem Essakly, Hakan Alakus, Reinhard Buettner, Axel M. Hillmer, Christiane Bruns, Wolfgang Schroeder, Thomas Zander, Heike Loeser, Florian Gebauer, Alexander Quaas

**Affiliations:** 1 Institute of Pathology, University of Cologne, Kerpener Str. 62, 50937 Cologne, Germany; 2 Department of General, Visceral and Cancer Surgery, University of Cologne, Cologne, Germany; 3 Department I of Internal Medicine, Center for Integrated Oncology (CIO)University of Cologne, Cologne, Germany

**Keywords:** SWI/SNF-complex, Loss-of-function, Oesophageal adenocarcinoma, TP53 loss, Heterogeneity

## Abstract

**Background:**

The SWI/SNF complex is an important chromatin remodeler, commonly dysregulated in cancer, with an estimated mutation frequency of 20%. ARID1A is the most frequently mutated subunit gene. Almost nothing is known about the other familiar members of the SWI/SNF complexes, SMARCA2 (BRM), SMARCA4 (BRG1) and SMARCB1 (INI1), in oesophageal adenocarcinoma (EAC).

**Methods:**

We analysed a large cohort of 685 patients with EAC. We used four different antibodies to detect a loss-of-protein of ARID1A BRM, BRG1 and INI1 by immunohistochemistry and correlated these findings with molecular and clinical data.

**Results:**

Loss of ARID1A, BRG1, BRM and INI1 was observed in 10.4, 3.4, 9.9 and 2% of EAC. We found a co-existing protein loss of ARID1A and BRM in 9.9% and of ARID1A and BRG1 in 2.2%. Patients with loss of ARID1A and TP53 wildtype EACs showed a shortened overall survival compared with AIRDA1A-positive tumours [median overall survival was 60.1 months (95%CI 1.2–139.9 months)] in patients with ARIDA-1A expression and 26.2 months (95%CI 3.7–19.1 months) in cases of ARIDA-1A loss (*p* = 0.044). Tumours with loss or expression of ARID1A and TP53 loss were not associated with a difference in survival. Only one tumour revealed high microsatellite instability (MSI-H) with concomitant ARID1A loss. All other ARID1A loss-EACs were microsatellite-stable (MSS). No predictive relevance was seen for SWI/SNF-complex alterations and simultaneous amplification of different genes (PIK3CA, KRAS, c-MYC, MET, GATA6, ERBB2).

**Conclusion:**

Our work describes, for the first time, loss of one of the SWI/SNF ATPase subunit proteins in a large number of adenocarcinomas of the oesophagus. Several papers discuss possible therapeutic interventions for tumours showing a loss of function of the SWI/SNF complex, such as PARP inhibitors or PI3K and AKT inhibitors. Future studies will be needed to show whether SWI/SNF complex-deficient EACs may benefit from personalized therapy.

## Background

Oesophageal cancer represents the eighth most common malignant tumour worldwide, with an estimated cancer burden of 455,800 cases and 400,200 deaths [1]. Men are 4 to 5 times more frequently affected than women. Histologically, the majority of oesophageal cancers are compartmentalized into squamous cell carcinoma (SCC) and adenocarcinoma (EAC). In recent decades, the incidence of SCC has decreased because of the long-term reduction in tobacco use and alcohol consumption, whereas the proportion of EAC has risen to approx. 33% [compare www.krebsdaten.de]. Most EACs arise within Barrett’s mucosa, which, among other things, is attributed to gastroesophageal reflux disease, obesity and low fruit and vegetable consumption. Despite the known tumour heterogeneity of oesophageal carcinomas, due to less reliable response molecules such as TUBB3, unselective preoperative radiochemotherapy or chemotherapy alone is usually the main therapeutic tool for many oesophageal cancer patients [2]. The overall survival has remained almost unchanged in recent years. For these reasons, new biomarkers are needed that can contribute to a better understanding of tumor biology in specific subgroups of the adenocarcinomas of the esophagus. This is also associated with the hope of being able to personalise the treatment of specific tumour subgroups. There is reason to believe that adenocarcinomas of the oesophagus that show a loss of function of the SWI/SNF complex may represent such a tumor subgroup, as discussed later. An important goal of this study is how often a loss of the SWI/SNF complex and its individual functional protein components is found in a very large collective of oesophageal adenocarcinomas and what other molecular characteristics this SWI/SNF-loss subgroup has.

SWitch/sucrose-nonfermenting (SWI/SNF) complexes, first described as transcriptional regulators of inducible and lineage-specific genes, are important members of the chromatin-remodelling family [3, 4]. Mammalian SWI/SNF’s consist of 12–15 subunits, of which an ATPase subunit [either SMARCA2 (BRM) or SMARCA4 (BRG1)] and three additional subunits [SMARCB1 (INI1), SMARCC1, SMARCC2] form the functional core. The complex has been shown to alter chromatin structure by repositioning, ejecting or exchanging nucleosomes and thereby plays an important role in many cellular processes, including transcription, cell cycle control, proliferation, differentiation and repair of DNA lesions [5–8]. Among different chromatin remodelers, SWI/SNF complexes, with an estimated mutation frequency of 20%, are the most commonly dysregulated epigenetic complexes in cancer, and *AT-rich interactive domain-containing protein 1A (ARID1A)* is the most frequently mutated gene subunit [9–12]. Mutations in *ARID1A* are generally inactivating and result in loss of ARID1A protein, which is detectable by immunohistochemistry. Loss of ARID1A expression has been found in a broad spectrum of human cancers, including gastric carcinoma (8–29%) and oesophageal adenocarcinoma (9–19%) [13–21].

Although no direct restoration of *ARID1A* is currently possible, loss of the tumour suppressor gene results in specific weak points in cancer cells that are suitable for therapy. Helming et al. identified ARID1B, a related homologue of ARID1A in the SWI/SNF complex, as the number one gene mainly required for the survival of ARID1A-mutant cancer cell lines and as a potential therapeutic target for ARID1A-mutant cancers [22]. In addition, a study in ovarian carcinomas showed that ARID1A deficiency – via interaction with MutS protein homolog 2 (MSH2) – leads to an impaired MMR phenotype in tumour cells that could be used for immunotherapy [23].

So far, little is known about the importance and possible heterogeneous distribution of ARID1A loss and its correlations to various other molecular changes at a very large collective of EAC. Almost nothing is known about the remaining ATPase subunit members (BRG, BRM1 and INI1) in EAC.

## Methods

### Patients

We analysed formalin-fixed, paraffin embedded material from 685 patients with EAC who underwent primary surgical resection or resection after neoadjuvant therapy between 1999 and 2016 at the Department of General, Visceral and Cancer Surgery, University of Cologne, Germany. The standard surgical procedure was laparotomic or laparoscopic gastrolysis and right transthoracic en bloc esophagectomy including two-field lymphadenectomy of mediastinal and abdominal lymph nodes. As described previously, reconstruction was performed by high intrathoracic esophagogastrostomy [24]. Patients with advanced oesophageal cancer (cT3, cNx, M0) obtained either preoperative chemoradiation or chemotherapy alone. All patients were monitored according to a standardized protocol. Follow-up examinations contained a extensive history, clinical evaluation, abdominal ultrasound, chest X-ray and additional diagnostic procedures as needed. Monitoring data were available for all patients. Patient characteristics are given in Table 1. As consequence of neoadjuvant radiochemo- or chemotherapy, there is a predominance of minor responders in the TMAs, defined as histopathological residual tumour of ≥10% [25]. Details are summarized in [2].

**Table 1:** Correlation of ARID1a, BRG1 and BRM expression for the entire patients cohort

	ARID1a expression						BRG1 expression						BRM expression					
		Negative		Positive		*p*-value		Negative		Positive		*p*-value		Negative		Positive		*p*-value
Total	560*	58	10.4%	502	89.6%		563*	19	3.4%	544	96.6%		578*	57	9.9%	521	90.1%	
Sex																		
Female	65	7	10.8%	58	89.2%	0.831	67	1	1.5%	66	98.5%	0.715	68	11	16.2%	57	83.8%	0.080
Male	495	51	10.3%	444	89.7%		496	18	3.6%	478	96.4%		510	46	9.0%	464	91.0%	
Age group																		
<65	185	17	9.2%	168	90.8%	0.399	186	4	2.2%	182	97.8%	0.239	191	20	10.5%	171	89.5%	0.199
>65	174	19	10.9%	155	89.1%		170	9	5.3%	161	94.7%		173	20	11.6%	153	88.4%	
pT stage																		
1	81	9	11.1%	72	88.9%	0.657	79	3	3.8%	76	96.2%	0.448	86	8	9.3%	78	90.7%	0.981
2	72	8	11.1%	64	88.9%		73	1	1.4%	72	98.6%		72	6	8.3%	66	91.7%	
3	385	41	10.6%	344	89.4%		388	13	3.4%	375	96.6%		398	41	10.3%	357	89.7%	
4	19	0	0%	19	100%		20	2	10%	18	90.0%		20	2	10.0%	18	90.0%	
Lymph node metastasis																		
pN0	217	31	14.3%	186	85.7%	0.086	216	8	3.7%	208	96.3%	0.952	227	20	8.8%	207	91.2%	0.651
pN 1	196	13	6.6%	183	93.4%		197	6	3%	191	97.0%		194	23	11.9%	171	88.1%	
pN 2	74	7	9.5%	67	90.5%		74	2	2.7%	72	97.3%		79	6	7.6%	73	92.4%	
pN 3	71	7	9.9%	64	90.1%		74	3	4.1%	71	95.9%		76	8	10.5%	68	89.5%	
Grading																		
G1	4	1	25%	3	75%	0.186	5	0	0%	5	100%	< 0.001	6	0	0%	6	100%	0.119
G2	218	16	7.3%	202	92.7%		217	3	1.4%	214	98.6%		221	20	9.0%	201	91.0%	
G3	167	22	13.2%	145	86,8%		172	10	5.8%	162	94.2%		177	24	13.6%	153	86.4%	

*Total numbers of patients do not add due to missing clinical data in some cases

#### Tumour samples

**Fig. 1:**
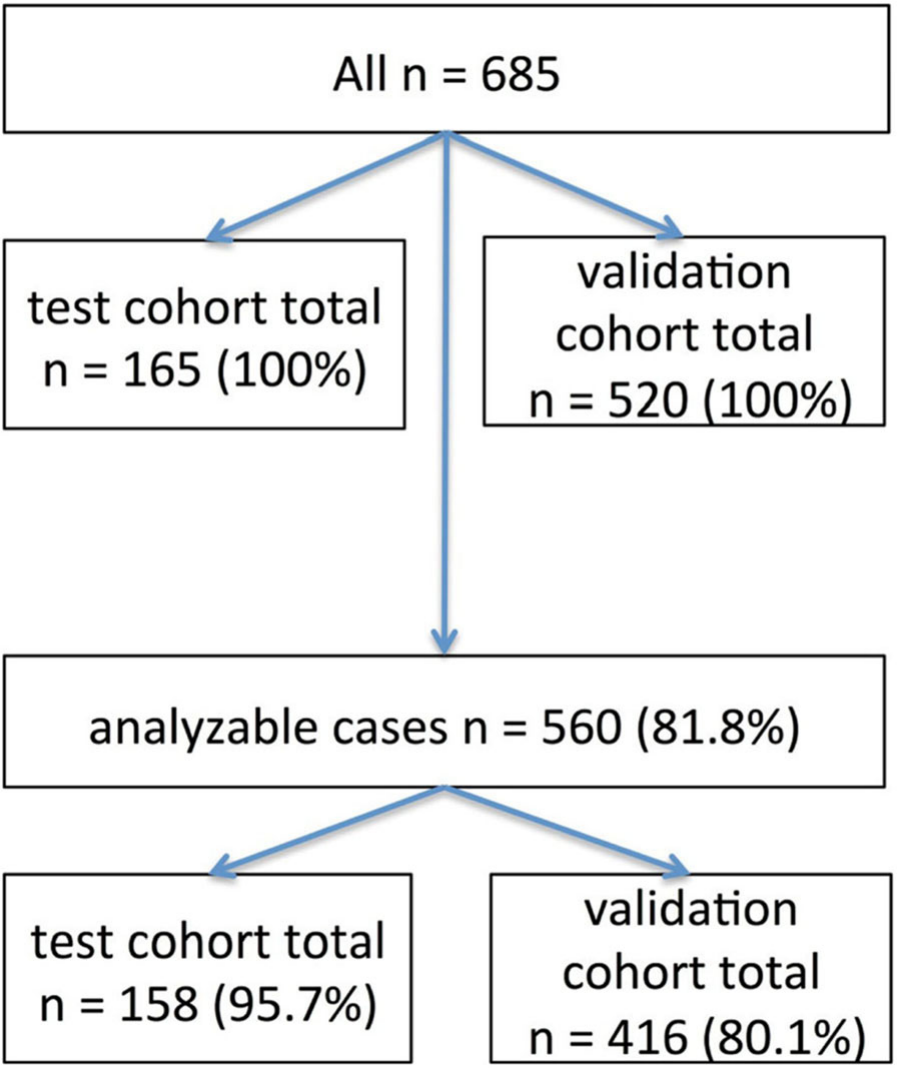
Flow diagram of patient cohorts

We used a multi-spot tissue microarray (TMA) with up to 12 tumour spots as a test cohort consisting of 165 patients with EAC (see Fig. 1).

In all, 148 patients (89%) of the test cohort did not receive any neoadjuvant treatment. We considered equally the tumour surface/centre and the tumour infiltration margin whenever possible. This multi-spot TMA is also suitable for detecting heterogeneous distribution of proteins within the tumour. In addition, we analysed 15 adenocarcinomas on whole slides with regard to possible heterogeneity. Furthermore, we created a single-spot TMA considering 520 additional patients as a validation cohort (685 patients in total).

For tissue microarray analysis (TMA), one tissue core from each tumour was punched out and transferred into a TMA recipient block. TMA construction was performed as previously described [26, 27]. In brief, tissue cylinders with a diameter of 1.2 mm each were punched from selected tumour tissue blocks using a self-constructed semi-automated precision instrument and embedded in empty recipient paraffin blocks. Four-micrometre sections of the resulting TMA blocks were transferred to an adhesive-coated slide system (Instrumedics Inc., Hackensack, NJ) for immunohistochemistry.

#### Immunhistochemistry

Immunohistochemistry (IHC) was performed on TMA slides. Lymphoid tissue served as an internal control. All markers showed a nuclear staining pattern. All details about the antibodies we used are listed in the supplement.

ARID1A-, BRG1-, BRM- and INI1 were analysed manually by two pathologists (A.Q. and S.S.). ARID1A-, BRG1-, BRM- and INI1 staining was assessed according to a two-tier scoring system (Score 0 and Score 1). A score of 0 associated with the loss of protein and defined as unequivocal clean absent staining in the nuclei of viable tumour cells for *ARID1A, BRG1-, BRM- or INI1* was interpreted as an underlying mutation, deletion or promotor alteration. Strong nuclear stainability of the surrounding non-tumour cells served as an internal control. Score 1 was determined as nuclear staining of tumour cells and interpreted as an intact, unmuted *ARID1-, BRG1-, BRM-* or *INI1* gene with regular protein expression. Discrepant results were resolved by consensus between the reviewers.

For analysis of ERBB2, membranous expression of HER2 in carcinoma cells was evaluated according to the criteria for biopsies as already described [28, 29].

The assessment of TP53 was carried out as already described [30].

We have analyzed all tumors for their DNA mismatch status for a previous publication (please compare [31]). For the current analyses we have again analyzed all tumors that showed an ARID1a loss and checked the DNA repair protein status with the recommended immunohistochemical markers (MLH1, MSH2, MSH6, PMS2) on whole tumor blocks. The methods used are also listed in detail in this publication [31].

#### Fluorescence in situ hybridization (FISH)

To determine the gene amplifications of *KRAS*, *PIK3CA*, *cMET*, *c-MYC* and *GATA6*, FISH was performed. All details about the FISH probes we used are listed in the supplement.

The strategy for detecting amplifications of *KRAS* followed the recommendations KRAS/CEN12 ratio ≥ 2.0 or KRAS extrachromosomal cluster signals [32].

PIK3CA gene amplification analysis was carried out according to the manufacturer’s protocol [33]. For PIK3CA of previous studies, PIK3CA/CEN3 ratio ≥ 2.0 or PIK3CA signals ≥5.0 define amplification.

MET amplification was defined as MET/CEP7 ratio ≥ 2.0 or a MET gene copy number > 4 [34].

Amplification of C-MYC was defined as gene copy cluster in > 50% of carcinoma cells or gene copy number (> 6) [35].

GATA6 amplification was defined as gene copy cluster in > 50% of carcinoma cells or gene copy number (> 6).

#### Data analysis and statistics

The current retrospective study was carried out with the approval of the Ethics Committee of the University of Cologne. Clinical data were collected prospectively according to a standardised protocol.

Including all types of mortality, prognosis was calculated beginning on the date of surgery. To describe survival distribution, Kaplan–Meier univariate analysis was used, and log-rank tests were used to evaluate survival differences. To analyse the effect of several risk factors on survival, we made use of the Cox proportional hazard regression with sequential backward elimination of the non-significant variables. Survival analysis and the multivariate cox-regression model were carried out on the entire cohort (test + validation cohort).

Descriptive analysis included the median with lower (LQ) and upper (UQ) quartiles for numeric variables (ordinal or asymmetric distribution), the frequency of nominal parameters, and the mean for numeric variables with a normal distribution. Univariate analysis was conducted for tables using chi-squared statistics or Fisher’s exact test if necessary. To determine whether there was a linear trend between the column number and the fraction of subjects in the top row, we made use of the Cochran–Armitage test for trend. The Mann–Whitney U test was employed to compare continuous variables. Significant differences between groups were defined as *p* < 0.05.

Statistical analyses were performed using the statistic program IBM SPSS v22.0 (IBM Corporation, New York, USA). MedCalc Statistical Software version 18.2.1 (MedCalc Software bvba, Ostend, Belgium; http://www.medcalc.org; 2018) was used for graphic presentation of the results. Details are summarized in [36].

## Results

### Clinico-pathological and patients´ characteristics

The test cohort (*n* = 165) consisted of 149 men (90.4%) and 16 women (9.7%), with a median age at the time of operation of 65.1 years (range 33–85 years). For the validation cohort, 520 additional patients were considered for analysis (685 patients with EAC in total). The median follow-up for the entire cohort was 57.7 months, with a calculated 5-year survival rate of 26.6%. Patient characteristics are given in Table 1. Out of 685 patients with EAC, 560 patients were finally analysable for ARID1A and INI1 (81.8%), 563 patients for BRG1 (82.2%) and 578 patients (84.4%) for BRM. Reasons for non-informative cases included lack of tissue samples or absence of unequivocal cancer tissue in the TMA spot. For the validation cohort only, 416 patients were analysable out of 520 (80.1%).

### Loss of ARID1A, BRG1, BRM and INI1 in the test cohort (*n* = 165) and heterogenous distribution

**Fig. 2:**
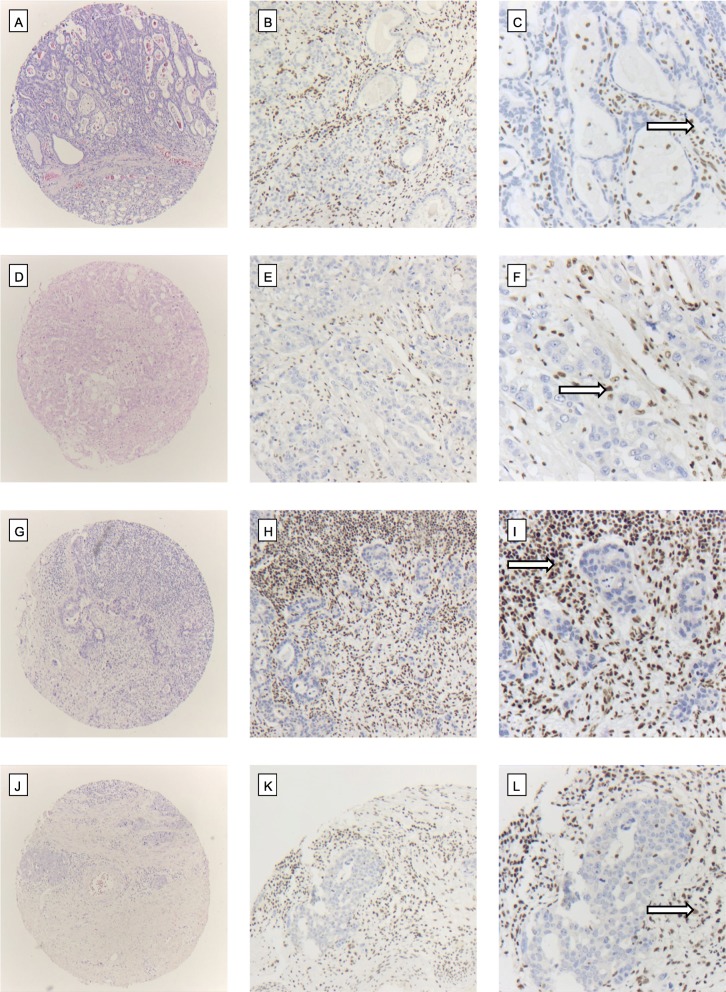
Protein-loss of ARID1a, BRM, BRG1 and INI1 in EAC. Top line: **(a)** EAC (HE, × 5) with loss of ARID1a expression **(b)** (IHC, × 10), **(c)** (IHC, × 20). Second line: **(d)** EAC (HE, × 5) with loss of BRM **(e)** (IHC, × 10), **(f)** (IHC, × 20). Third line: **(g)** EAC (HE, × 5) with loss of BRG1 **(h)** (IHC, × 10), **(i)** (IHC, × 20). Bottom line: **(j)** EAC (HE, × 5) with loss of INI1 **(k)** (IHC, × 10), **(l)** (IHC, × 20). Strong nuclear staining of the surrounding non-tumor cells served as internal control (arrow)

On the test cohort TMA between 147 and 149 cases were analysable, respectively depending on the analysed protein. We found losses of ARID1A, BRM, BRG1 and INI1 in 9,5%, 11,5%, 4,7 and 0.6% of cases. In 57 tumours we found a co-existing protein loss of ARID1A and BRM (9.9%) and in 15 samples (2.2%) a protein loss of ARID1A and BRG1 (compare Fig. 2). A coexisting loss considering INI1 was not detectable due to the low number of INI1 losses in the entire cohort (only one tumour), baseline characteristics are displayed in Table 2.

**Table 2:** Correlation of ARID1a, BRG1 and BRM expression for the test corhort

		ARID1a expression							BRG1 expression							BRM expression					
			Negative		Positive		*p*-value			Negative		Positive		*p*-value			Negative		Positive		*p*-value
Total	147*		14	9.5%	133	90.5%		149*		7	4.7%	142	95.3%		148*		17	11.5%	131	88.5%	
Sex																					
Female	13	8.8%	1	7.7%	12	92.3%	1.000	14	9.4%	0	0.0%	14	100.0%	1.000	14	9.5%	2	14.3%	12	85.7%	0.664
Male	134	13.2%	13	9.7%	121	90.3%		135	90.6%	7	5.2%	128	94.8%		134	90.5%	15	11.2%	119	88.8%	
Age group																					
<65	63	46.3%	5	7.9%	58	92.1%	0.771	65	47.1%	2	3.1%	63	96.9%	0.447	65	47.4%	7	10.8%	58	89.2%	0.796
>65	73	53.7%	8	11.0%	65	89.0%		73	52.9%	5	6.8%	68	93.2%		72	52.6%	9	12.5%	63	87.5%	
pT stage																					
1	21	14.4%	2	9.5%	19	90.5%	0.583	21	14.2%	1	4.8%	20	95.2%	0.107	21	14.3%	3	14.3%	18	85.7%	0.496
2	13	8.9%	0	0.0%	13	100.0%		13	8.8%	0	0.0%	13	100.0%		13	8.8%	0	0.0%	13	100.0%	
3	109	74.7%	12	11.0%	97	89.0%		111	75.0%	5	4.5%	106	95.5%		110	74.8%	14	12.7%	96	87.3%	
4	3	2.1%	0	0.0%	3	100%		3	2.0%	1	0.0%	2	100%		3	2.0%	0	0.0%	3	100.0%	
Lymph node metastasis																					
pN0	54	37.0%	6	11.1%	48	88.9%	0.656	54	36.5%	3	5.6%	51	94.4%	0.832	54	36.7%	5	9.3%	49	90.7%	0.574
pN 1	71	48.6%	5	7.0%	66	93.0%		71	48.0%	3	4.2%	68	95.8%		70	47.6%	8	11.4%	62	88.6%	
pN 2	10	6.8%	1	10.0%	9	90.0%		10	6.8%	0	0.0%	10	100.0%		10	6.8%	1	10.0%	9	90.0%	
pN 3	11	7.5%	2	18.2%	9	81.8%		13	8.8%	1	7.7%	12	92.3%		13	8.8%	3	23.1%	10	76.9%	
Grading																					
G1	0	0.0%	0	0.0%	0	0.0%	0.095	1	0.8%	0	0.0%	1	100.0%	0.901	1	0.8%	0	0.0%	1	100.0%	0.123
G2	84	68.9%	5	6.0%	79	94.0%		84	67.7%	3	3.6%	81	96.4%		83	67.5%	8	9.6%	75	90.4%	
G3	38	31.1%	6	15.8%	32	84.2%		39	31.5%	2	5.1%	37	94.4%		39	31.7%	9	23.1%	30	76.9%	

*Total numbers of patients do not add due to missing clinical data in some cases

### Loss of ARID1A, BRG1, BRM and INI1 in the validation cohort and test cohort

The validation and test cohort showed no significant differences in the clinical and pathological base line characteristics with a similar distribution with respect to sex, patients age, tumor stage (pT), lymph node metastasis (pN) and grading. Loss of ARID1A was observed in 10.4% (*n* = 43), loss of BRG1 in 3.4% (*n* = 14), loss of BRM in 9.9% (*n* = 41) and loss of INI1 in 2% (n = 1) of cases. Correlation with clinical and pathological staging is shown in Table 1. Except an association between BRG1 expression and grading, there were no correlations detectable in cross-table analysis between loss of any of the analysed complex proteins and clinical or pathological parameters. Due to only 1 patient having loss of INI1 expression in the entire cohort, cross-table analysis (chi-square test) was not applicable.

For the entire patients cohort, loss of ARID1A was observed in 58 patients (10.3%), BRG1 in 19 patients (3.4%), BRM *n* = 57 (10.0%) and INI1 *n* = 1 (1.9%). Cross-table analysis and Table 2 was based on the entire patients cohort.

### Heterogeneity of ARID1A, BRG1, BRM and INI1 loss

**Fig. 3:**
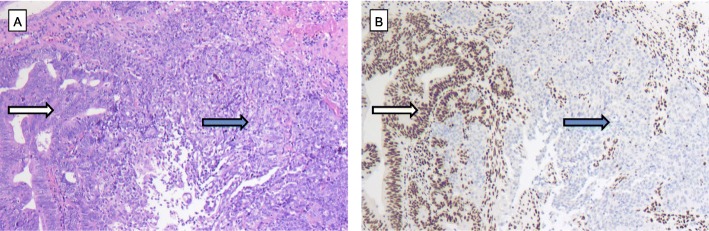
Heterogeneity of ARID1a loss. **(a)** EAC (HE, × 10) with **(b)** immunohistochemically preserved protein expression in the left tumor area (white arrow) showing gland-forming carcinoma cells mixed with portions that show complete ARID1a loss (blue arrow) in solid growing tumor cells (IHC, × 10)

We defined heterogeneity as tumours with preserved protein expression and areas with complete protein loss. Heterogeneity of protein loss was low in the entire cohort. We observed heterogeneous distribution of ARID1A in 4.8% of the tumours, heterogeneous distribution of BRM in 9.6% of cases and heterogeneous distribution of BRG1 in 0.6% of the EACs. The single INI1-deficient carcinoma showed homogeneous negativity within the entire tumour area (compare Fig. 3).

### Correlation with other molecular changes

**Fig. 4:**
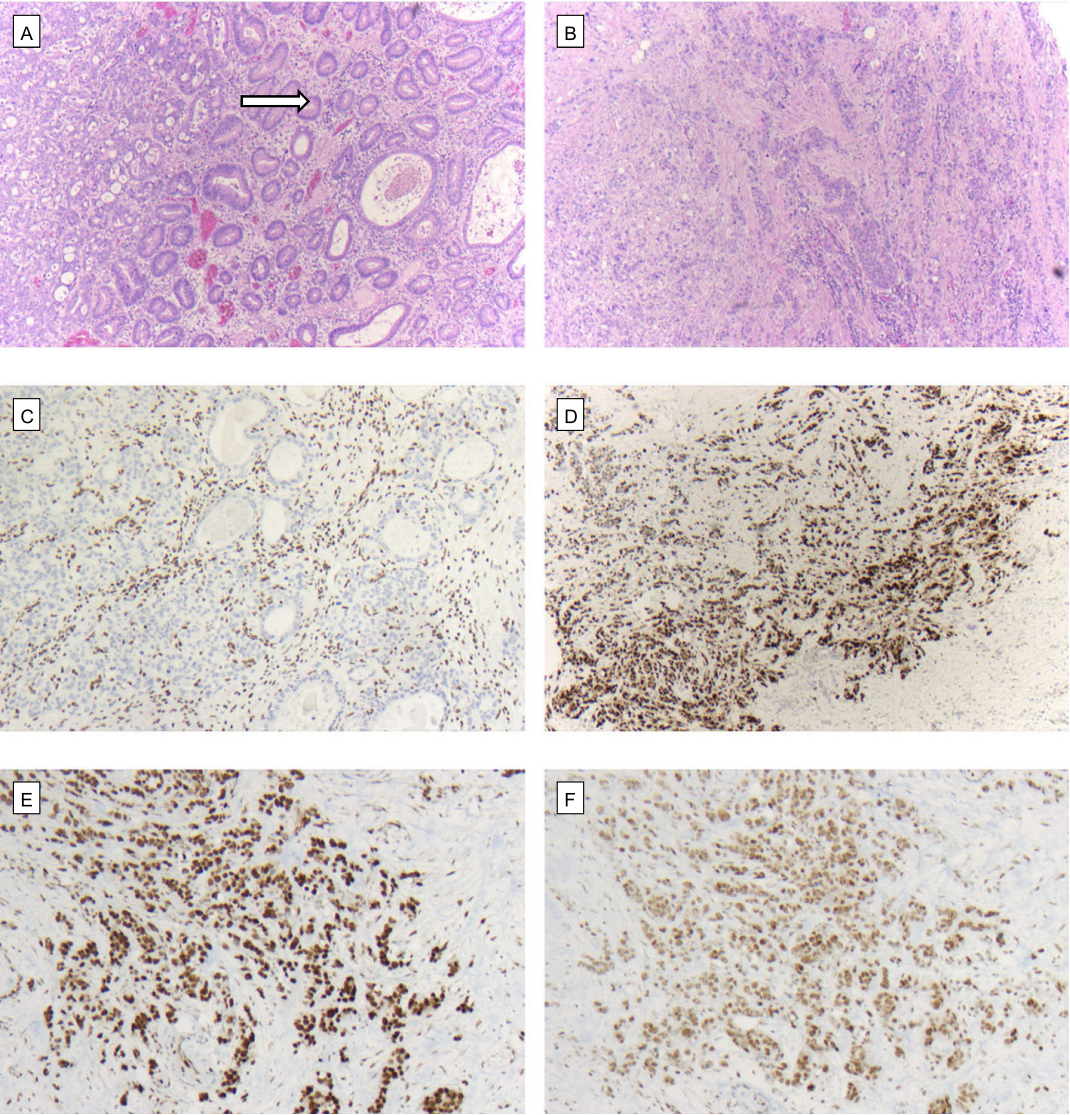
Loss of ARID1a in TP53 loss, microsatellite-stable (MSS) EAC. **(a + b)** EAC of the gastroesophageal junction (arrow highlights gastric mucosa; HE, × 5) with **(c)** loss of ARID1a (IHC, × 10), **(d)** strong nuclear positivity of TP53 as a sign of TP53 loss (IHC, × 10) and **(e)** nuclear positivity of the mismatch-repair-proteins MSH6 (IHC, × 10) and **(f)** MLH1 (IHC, × 10)

Analysis of the correlation with several other molecular changes in our EAC collective was performed considering TP53 loss, *ERBB2* amplification and Her2/neu protein expression, *MET* amplification, c-MYC amplification, *KRAS* amplification, *PIk3CA* amplification, *GATA6* amplification and loss of DNA-mismatch-repair protein (MLH1, PMS2, MSH6, MSH2). *TP53* loss and *ERBB2* amplification were available in 366 patients, and all other molecular changes were available for 520 patients (compare Fig. 4 and Table 3). A correlation was detectable between loss of BRM and *MET* amplification. A correlation between ARID1A loss and any of the analysed molecular markers was not observed, including loss of DNA-mismatch repair proteins (only one MSI-H tumour with a loss of MLH1 and PMS2 expression demonstrated an additional ARID1A loss).

**Table 3:** Correlation of ARID1a, BRG1 and BRM1 expression with molecular tumor data

		ARID1a expression					BRG1 expression					BRM expression				
		Negative		Positive			Negative		Positive			Negative		Positive		
		No.	%	No.	%	*p* value	No.	%	No.	%	*p* value	No.	%	No.	%	*p* value
MET amplification	none	45	9.9%	408	90.1%	0.642	18	4.0%	433	96.0%	1.00	44	9.6%	414	90.4%	0.002
	amplified	2	14.3%	12	85.7%		0	0.0%	15	100.0%		6	40.0%	9	60.0%	
C-myc amplification	none	44	10.5%	376	89.5%	0.099	17	4.0%	406	96.0%	0.709	45	10.5%	384	89.5%	0.491
	amplified	2	3.4%	56	96.6%		1	1.8%	56	98.2%		4	6.9%	54	93.1%	
KRAS amplification	none	39	9.7%	364	90.3%	0.839	17	4.2%	384	95.8%	0.548	46	11.3%	360	88.7%	0.037
	amplified	7	8.0%	80	92.0%		2	2.2%	87	97.8%		4	4.3%	90	95.7%	
GATA6 amplification	none	44	10.4%	381	89.6%	0.070	19	4.5%	405	95.5%	0.240	45	10.5%	384	89.5%	0.631
	amplified	1	2.1%	46	97.9%		0	0.0%	47	100.0%		6	12.2%	43		
PIK3CA amplification	none	44	10.7%	368	89.3%	1.000	15	3.6%	400	96.4%	0.236	47	11.2%	371	88.8%	0.417
	amplified	2	8.3%	22	91.7%		2	8.3%	22	91.7%		3	12.5%	21	87.5%	
ERBB2 amplification	none	33	10.7%	275	89.3%	0.596	13	4.2%	297	95.8%	0.381	35	11.3%	276	88.7%	1.000
	amplified	3	7.0%	40	93.0%		0	0.0%	43	100%		4	9.5%	38	90.5%	
TP53 mutation	negative	16	10.5%	136	89.5%	0.594	7	4.6%	146	95.4%	0.410	19	12.8%	130	87.2%	0.304
	mutated	19	8.9%	195	91.1%		6	2.9%	200	97.1%		20	9.2%	197	90.8	

A correlation between loss of ARID1A and the histological subtype was also carried out. We were not able to detect a correlation between morphological aspects of the tumour or grading and ARID1A status (data not shown).

### Loss of SWI/SNF complex proteins and oncological long-term prognosis

**Fig. 5:**
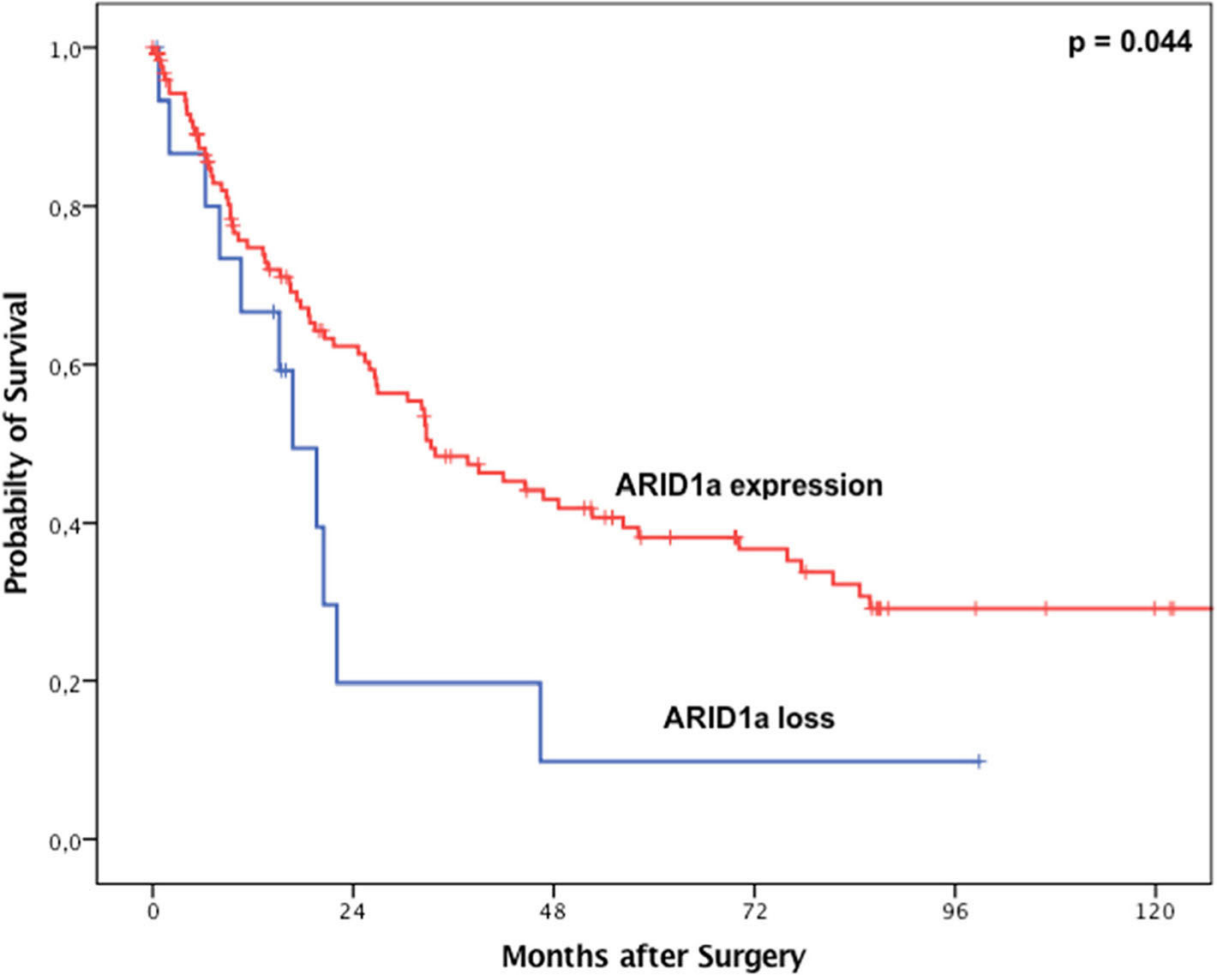
Kaplan-Meier survival analysis (log-rank test) for the patient cohort with TP53 wild-type EACs and ARID1a proficient versus deficient expression. Significant survival differences between patients with TP53 non-mutated (wild-type) EACs and ARID1a expression (median overall-survival 60.1 months (95% confidence interval (95%CI) 1.2–139.9 months) compared to TP53 wild-type EACs showing an ARID1a loss (median overall-survival 26.2 months (95%CI 3.7–19.1 months), *p* = 0.044)

**Fig. 6:**
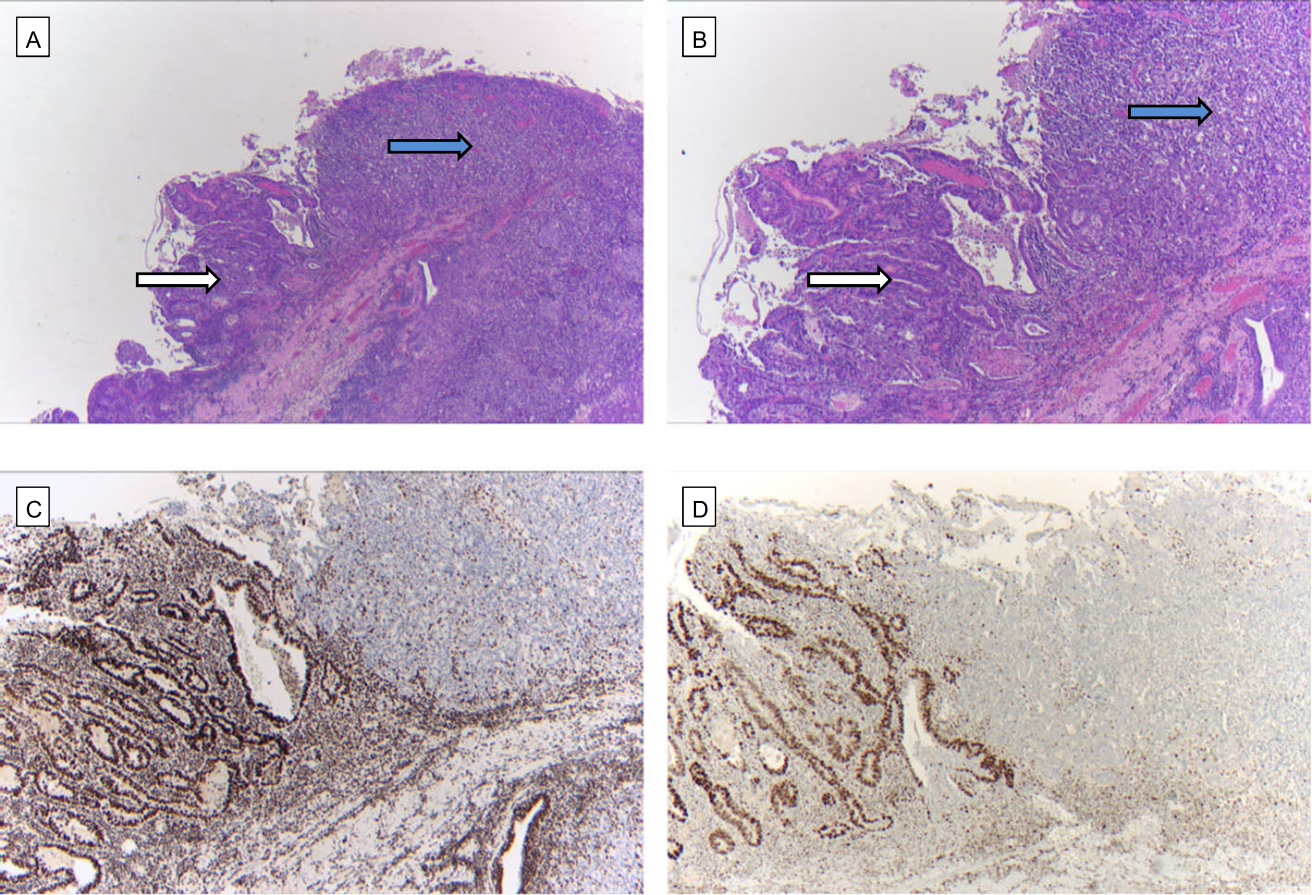
Loss of ARID1a in microsatellite-instable (MSI-H) EAC. **(a + b)** Barrett’s adenocarcinoma (white arrow highlights Barrett’s mucosa with high grade dysplasia, blue arrow highlights highly inflamed and poorly cohesive Barrett’s carcinoma; HE, 2,5x; HE, × 5;) with **(c)** loss of ARID1a in Barrett’s carcinoma and strong nuclear positivity in high grade dysplasia (IHC, × 5) and **(d)** loss of the mismatch-repair-protein MLH1 in Barrett’s adenocarcinoma (IHC, × 5)

For the entire cohort, loss of ARID1A, BRM, BRG1 or INI1 was not correlated with shortened overall survival in Kaplan-Meier survival analysis. However, patients with loss of ARID1A and wildtype TP53 showed a shortened overall survival compared with ARID1A expressing patients (Fig. 5). In tumours with wildtype TP53, median overall survival was 60.1 months [95% confidence interval (95%CI) 1.2–139.9 months] in patients with ARID1A expression and 26.2 months (95%CI 3.7–19.1 months) in the case of loss of ARID1A (*p* = 0.044). Tumours with ARID1A loss or expression and TP53 loss were not associated with a difference in survival. Only one tumour showed high microsatellite instability (MSI-H) with concomitant ARID1A loss (compare Fig. 6). All other EACs with ARID1A loss were microsatellite-stable (MSS).

### Multivariate analysis

In a multivariate cox- regression model, only the UICC stage was found as independent prognosticator (see Table 4).

**Table 4:** Multivariate cox-regression model*

	Hazard ratio	95% confidence interval		*p* value
		Lower	Upper	
SEX (male vs. female)	1.731	0.956	3.133	0.07
Agegroup (< 65 yrs vs. > 65 yrs)	1.322	0.994	1.76	0.055
Tumor stage (pT1/2 vs. pT3/4)	1.084	0.706	1.666	0.712
Lymph node metastastasis (pN0 vs pN+)	1.108	0.676	1.814	0.685
UICC Stage (UICC Stage I/II vs. III/IV)	1.965	1.506	2.562	0
ARID1A (loss vs. expression)	0.734	0.465	1.158	0.184
BRG1 (loss vs. expression)	0.633	0.294	1.363	0.243
BRM (loss vs. expression)	1.27	0.789	2.045	0.325

*due to only one patient with loss of INI. INI was not considered in the multivariate cox-regression model

## Discussion

This is the first study to investigate the importance of protein loss of various components of the ATPase subunit of the SWI-SNF complex in a very large group of oesophageal adenocarcinomas.

In the few studies that exist on oesophageal adenocarcinoma on this subject, individual components (ARID1A loss) of the SWI/SNF complex were analysed. Recently, ARID1A loss was detected in 10% of the 120 oesophageal adenocarcinomas examined in a work using a similar methodology [20]. Thus, our study describes identical results of loss of ARID1A expression in EAC. However, based on immunohistochemical analysis, we cannot determine precisely the underlying cause of protein loss. Nonetheless, the loss of protein detection is clearly associated with a loss of function of the protein in vivo; therefore, the immunohistochemistry used here is an effective method of deducing an underlying genomic alteration or a dysregulation of promoter activity. A functional loss of ARID1A has been increasingly scientifically studied in recent years. Thus, in addition to its function as a chromatin remodeler, ARID1A is regarded as a tumour suppressor gene [17, 37].

Within the gastrointestinal tract, the importance of ARID1a loss in gastric carcinoma and colon carcinoma has been described. In particular, an association with microsatellite instability (MSI-H) was described in both tumour entities [38] and additionally in gastric carcinoma, an association with the EBV subtype [39]. We were able to find a link to MSI-H and ARID1A loss in our cohort in only one tumour. This is mainly related to the exceedingly rare MSI-H findings in EAC. We and others showed that MSI-H in EAC is rare (< 1%) in comparison with gastric carcinoma (up to 21%) or colon carcinoma (about 13%) [31, 40, 41]. Interestingly, in this case, high-grade dysplastic Barrett’s mucosa (high-grade intraepithelial neoplasia), marginal to the invasive tumour, revealed expression of MLH1, PMS2 and ARID1A (compare Fig. 6). This suggests that the putative underlying MLH1 promoter methylation is a very late event that occurs just prior to invasive growth behaviour of the tumour. This has already been shown in gastric carcinoma. In this case, the loss of ARID1A is also associated as a late event with MLH1/PMS2 failure and was related to a TP53 loss.

In MSI-H tumours, alteration of ARID1A and other genes of the SWI/SNF complex could only be an epiphenomenon, since this tumour subtype already has many mutations due to its DNA repair defect, so that ARID1A alterations could only be passenger mutations without biological relevance of their own. According to our results, this does not apply to adenocarcinomas of the oesophagus. We can thus confirm the results of the only other study that has dealt with ARID1A loss in EAC thus far. Apparently, mutually exclusive mutations of ARID1A and TP53 are not present in EAC. Our data suggest that alterations in ARID1A may be a relevant event in EAC. In this context it is interesting to note that alterations of ARID1A are already found in precursor lesions of EAC (Barrett’s dysplasia), and here we show a mostly homogeneous loss of ARID1A in the tumour [15]. In the group of tumours with wildtype TP53, we could prove a significantly worse prognosis in EACs with ARID1A loss. There is strong evidence that tumours with altered ARID1A represent a biologically relevant tumour subgroup of EACs. Future studies are needed to investigate whether this subgroup can be specifically therapeutically targeted. Several papers discuss possible therapeutic interventions, for example, by targeting the role of ARID1A in regulating DNA damage checkpoints at double strand breaks, which sensitize tumour cells to PARP inhibitors [23, 42]. Furthermore, ARID1A mutations are commonly associated with protein kinase B (PKB, AKT) phosphorylation and, consequently, with phosphatidylinositol 3-kinase (PI3K) pathway activation [43, 44]. Loss of ARID1A expression was found to increase the response of cancer cells to PI3K and AKT inhibitors significantly [45]. Other publications identified the antioxidant glutathione (GSH), the glutamate-cysteine ligase synthetase catalytic subunit (GCLC), a rate-limiting enzyme for GSH synthesis, and EZH2, a histone-lysine N-methyltransferase enzyme that participates in histone methylation and transcriptional repression, as novel treatment options for cancers involving ARID1A mutations [46, 47].

Nearly nothing is known about the functional relevance of the other investigated proteins of the ATPase subunit (BRM, BRG1, INI1) in oesophageal carcinoma. Only one study of gastrointestinal tract tumours has shown a connection to undifferentiated carcinomas thus far [48]. Recently it has been shown that the loss of BRG1 and BRM in other solid tumors (ovar and lung) benefit from therapy with Bromodomain inhibitors (BETi). The authors of this study come to the conclusion „these findings address an unmet clinical need by identifying loss of SMARCA4/A2 as biomarkers of hypersensitivity to BET-inhibitors “[49].

Interestingly in our study, there was also a significant correlation between BRG1 silencing with EAC grading (*p* < 0.001). On the other hand, loss of BRM and INI1 was not correlated with EAC grading. Furthermore a correlation between a BRM loss and a MET amplification could be detect. Unfortunately, even an extensive literature search did not provide any information on the underlying mechanisms. Loss of BRG1 or INI1 was not associated with other molecular changes. BRM, BRG1 or INI1 silencing did not correlate with shortened overall survival in Kaplan-Meier survival analysis or morphological aspects.

## Conclusion

In conclusion, this study describes for the first time the importance of a failure of the SWI/SNF ATPase subunit ARID1A in a large number of adenocarcinomas of the oesophagus. In addition to ARID1A, we observed a significant amount of protein loss of BRM, to a lesser extent, of BRG1 and, very rarely, of INI1. In EAC, we did not observe an important association with microsatellite instability (only one case), in contrast to gastric carcinomas. Within the group of *TP53* wild type tumours, ARID1A-negative tumours show a significantly worse prognosis. No predictive relevance was seen for SWI/SNF-complex alterations and simultaneous amplification of different genes (*PIK3CA, KRAS, c-MYC, MET, GATA6, ERBB2)*.

The majority of tumours show a homogeneous loss of these proteins within the tumour. Future studies will be needed to show whether EACs that show a loss of function of the SWI/SNF complex can benefit from personalized therapies.

## Abbreviations

ARID1A: AT-rich interactive domain-containing protein 1A
EAC: Adenocarcinoma
GCLC: Glutamate-cysteine ligase synthetase catalytic subunit
GSH: Glutathione
MSH2: MutS protein homolog 2
MSI-H: Microsatellite instability
PI3K: Phosphatidylinositol 3-kinase
SCC: Squamous cell carcinoma
SWI/SNF: SWitch/sucrose-nonfermenting
